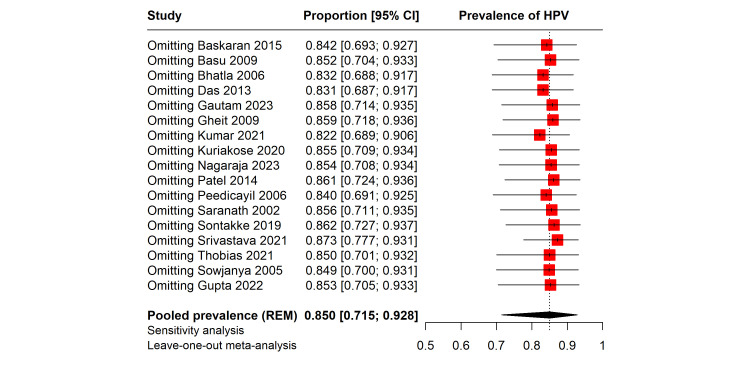# Prevalence of human papilloma virus among cervical cancer patients in India: A systematic review and meta-analysis

**DOI:** 10.1097/MD.0000000000038827

**Published:** 2024-08-02

**Authors:** Prakasini Satapathy, Mahalaqua Nazli Khatib, Ahmad Neyazi, Laila Qanawezi, Saida Said, Shilpa Gaidhane, Quazi Syed Zahiruddin, Sarvesh Rustagi, Marwan Al-Hajeili, Altaf A. Abdulkhaliq, Ahmed Alsayyah, Hayam A. Alrasheed, Maha F. Al-Subaie, Nawal A. Al Kaabi, Ali A. Rabaan

**Affiliations:** a Center for Global Health Research, Saveetha Medical College and Hospital, Saveetha Institute of Medical and Technical Sciences, Saveetha University, Chennai, India; b Medical Laboratories Techniques Department, AL-Mustaqbal University, Hillah, Babil, Iraq; c Division of Evidence Synthesis, Global Consortium of Public Health and Research, Datta Meghe Institute of Higher Education, Wardha, India; d Herat Maternity Hospital, Herat Regional Hospital, Herat, Afghanistan; e One Health Centre (COHERD), Jawaharlal Nehru Medical College, Datta Meghe Institute of Higher Education, Wardha, India; f South Asia Infant Feeding Research Network (SAIFRN), Division of Evidence Synthesis, Global Consortium of Public Health and Research, Datta Meghe Institute of Higher Education, Wardha, India; g School of Applied and Life Sciences, Uttaranchal University, Dehradun, Uttarakhand, India; h Department of Medicine, College of Medicine, King Abdulaziz University, Jeddah, Saudi Arabia; i Department of Biochemistry, Faculty of Medicine, Umm Al-Qura University, Makkah, Saudi Arabia; j Department of Pathology, College of Medicine, Imam Abdulrahman Bin Faisal University, Dammam, Saudi Arabia; k Department of Pharmacy Practice, College of Pharmacy, Princess Nourah Bint Abdulrahman University, Riyadh, Saudi Arabia; l Research Center, Dr. Sulaiman Alhabib Medical Group, Riyadh, Saudi Arabia; m College of Medicine, Alfaisal University, Riyadh, Saudi Arabia; n College of Medicine and Health Science, Khalifa University, Abu Dhabi, United Arab Emirates; o Sheikh Khalifa Medical City, Abu Dhabi Health Services Company (SEHA), Abu Dhabi, United Arab Emirates; p Molecular Diagnostic Laboratory, Johns Hopkins Aramco Healthcare, Dhahran, Saudi Arabia; q Department of Public Health and Nutrition, The University of Haripur, Haripur, Pakistan.

**Keywords:** cervical carcinoma, human papillomavirus, systematic review, vaccination

## Abstract

**Background:**

Cervical cancer, predominantly caused by the human papillomavirus (HPV), is a major health challenge in India, with high morbidity and mortality rates. Given India’s vast geographic and socio-economic diversity, understanding regional variations in HPV prevalence is crucial for developing targeted and effective public health interventions. This systematic review and meta-analysis were conducted to elucidate the prevalence of HPV among cervical cancer patients in India.

**Methods:**

A literature search was executed across PubMed, EMBASE, and Web of Science up to December 07, 2023. Observational studies reporting HPV prevalence among cervical cancer patients in India are included. A Modified Newcastle-Ottawa scale was used for quality assessment. A random-effects meta-analysis was used to determine pooled HPV prevalence, and heterogeneity was evaluated using the I² statistic. Subgroup and sensitivity analyses were performed to assess result stability and investigate heterogeneity sources. All statistical analyses were performed using R software version 4.3.

**Results:**

The meta-analysis included 17 studies with a total of 2529 cervical cancer cases, of which 1977 were HPV-positive. The pooled HPV prevalence was 85% (95% CI: 71–92%), with substantial heterogeneity (I² = 94%). Subgroup analysis by geographic zones showed notable differences: South (88%, 95% CI: 76–95%), North (73%, 95% CI: 1–100%), East (99%, 95% CI: 1–100%), Central (71%, 95% CI: 54–84%), and West (77%, 95% CI: 0–100%). Sensitivity analysis demonstrated the consistency of the results, and a reanalysis, excluding influential studies, yielded a prevalence of 82% (95% CI: 67–91%).

**Conclusion:**

Our analysis reveals a high prevalence of HPV in cervical cancer patients in India, with significant regional variations. The observed heterogeneity highlights the complexity of HPV epidemiology in India and necessitates further research to explore underlying causes and regional characteristics. Future studies should aim to expand geographic representation and deepen understanding of the factors contributing to the variability in HPV prevalence.

## 1. Introduction

Cervical cancer is a global health concern, currently ranked as the fourth most prevalent cancer in women both in terms of new cases and mortality rates.^[1,2]^ The global trend shows a gradual decrease in the incidence of cervical cancer, a positive development likely attributed to increased awareness, better screening practices, and the introduction of the human papillomavirus (HPV) vaccine.^[1,3]^ However, the incidence of cervical cancer is still heavily influenced by socio-economic factors and the prevalence of HPV, particularly types 16 and 18, which are responsible for approximately 80% of all cervical cancer cases.^[4,5]^ This strong association with HPV emphasizes the importance of effective prevention methods, notably vaccination and regular screening programs, which play crucial roles in mitigating this public health challenge.^[6]^

The burden of HPV-related cancers is particularly high, with cervical cancer constituting 87.6% of such cases in females.^[7]^ In India, cervical cancer represents a more acute problem, being the second most common cancer among women. It accounts for 10% of all female cancers in the country, significantly contributing to cancer-related morbidity and mortality among Indian women.^[8]^ Annually, India records approximately 132,000 new cases and 74,000 deaths due to cervical cancer, representing nearly a third of the global cervical cancer deaths.^[1]^ Additionally, more than 80% of sexually active women in India are estimated to acquire genital HPV by the age of 50.^[9]^ Addressing cervical cancer in India is challenged by several factors, including difficulties in cancer surveillance. This includes inadequate follow-up data and the lack of a comprehensive cause-of-death registration system. Despite these challenges, the Indian government has shown a commitment to combating cervical cancer, aligning with the World Health Organization’s global target of eliminating cervical cancer as a public health problem.^[8–10]^

Cervical cancer significantly strains India’s health system, necessitating a thorough investigation into the prevalence of HPV among those affected. Prior studies conducted across India’s varied geographical regions have reported notable variability in HPV prevalence. This systematic review and meta-analysis aims to synthesize and critically evaluate the available data to provide a comprehensive assessment of HPV prevalence throughout the different regions of India. By identifying regional disparities and gaps in existing research, this analysis supports the development of more precise, region-specific public health strategies for HPV vaccination and cervical cancer screening.

## 2. Methods

We adhered to the Preferred Reporting Items for Systematic Reviews and Meta-Analyses guidelines for conducting this systematic review and meta-analysis,^[11]^ as detailed in Table S1, Supplemental Digital Content, http://links.lww.com/MD/N141. For the screening of articles and data extraction, we employed the semiautomated software Nested Knowledge, which improved both efficiency and accuracy. The protocol for this systematic review is registered with PROSPERO.

### 2.1. Selection criteria

Original observational studies that reported the number of HPV-positive samples among cervical cancer patients were included in this study. Only confirmed HPV cases, tested using any standard criteria, were considered. Studies where the total population number or the number or proportion of HPV infection was not explicitly given were excluded. This study includes only adults (over 18 years of age). Case reports, case series, reviews, nonhuman studies, and commentaries were excluded from the systematic review. Articles available only in the English language were included. Studies from India or those including a population from India were specifically included. There were no restrictions on the setting of the study, whether it was at the hospital level or community level research. The detailed inclusion criteria are given in Table S2, Supplemental Digital Content, http://links.lww.com/MD/N142.

### 2.2. Literature search

An electronic search was conducted across multiple databases, including PubMed, EMBASE, and Web of Science, from their inception up to December 07, 2023. The initial search results were independently reviewed by 2 of the authors to verify their completeness and accuracy. We utilized the Nested Knowledge software for automatic searching in PubMed. No filters were applied regarding article type, publication year, or language of publication. Keywords and MeSH terms related to “HPV” and ‘Cervical Cancer’ were used. The detailed search strategy is available in Table S3, Supplemental Digital Content, http://links.lww.com/MD/N143.

### 2.3. Screening of articles

The Nested Knowledge software was utilized for deduplication and assistance in the screening of the searched articles. The screening of articles was conducted in 2 steps. The initial step involved primary screening through the review of titles and abstracts, followed by full-text screening for a detailed examination of the entire articles. Two independent reviewers carried out the screening process. Conflicts between the reviewers concerning article eligibility were resolved through consultation with a third reviewer.

### 2.4. Data extraction and quality assessment

We utilized the “tagging” feature of the Nested Knowledge web software to aid in data extraction. The “tags” were then transferred to Microsoft Excel. Data extraction was performed by 3 reviewers, and a fourth reviewer cross-checked all the extracted data to ensure accuracy. The extracted data includes the authors’ names, year of publication, sample size, number of HPV positive cases, the method used for HPV detection, and the region of the study. The quality assessment of the included studies was performed using a modified Newcastle-Ottawa Scale.^[12]^

### 2.5. Statistical analysis

A meta-analysis was performed to determine the pooled prevalence of HPV among cervical cancer patients. This involved comparing the number of HPV-positive samples to the total number of cervical cancer patients using a random-effects model. Heterogeneity among the studies was quantitatively assessed using the I² statistic.^[13–15]^ This statistic offers an estimation of the proportion of total variation among studies attributed to heterogeneity rather than random chance. An I² value of 0% signifies the absence of observed heterogeneity, whereas higher values indicate a growing level of heterogeneity, with 25% considered low, 50% moderate, and 75% high.^[16]^ We calculated the tau-squared value using maximum likelihood estimation to gain additional insights into heterogeneity.^[17,18]^ In cases where substantial heterogeneity (I² > 50%) was detected, a subgroup analysis was conducted to explore potential sources of this variation. These analyses were based on different characteristics, such as study design, geographic region, and method of HPV detection. Sensitivity analysis was also performed to assess the robustness of the meta-analysis results. This involved sequentially excluding individual studies to observe the impact on the overall effect size and heterogeneity. Publication bias was evaluated using funnel plots and further quantitatively assessed using Egger regression test. A *P*-value below .05 was typically regarded as statistically significant. A symmetric funnel plot and a nonsignificant Egger test would suggest a low likelihood of publication bias. All statistical analyses were performed using R software Version 4.3.^[19–21]^

## 3. Results

### 3.1. Literature search

**Figure 1.**
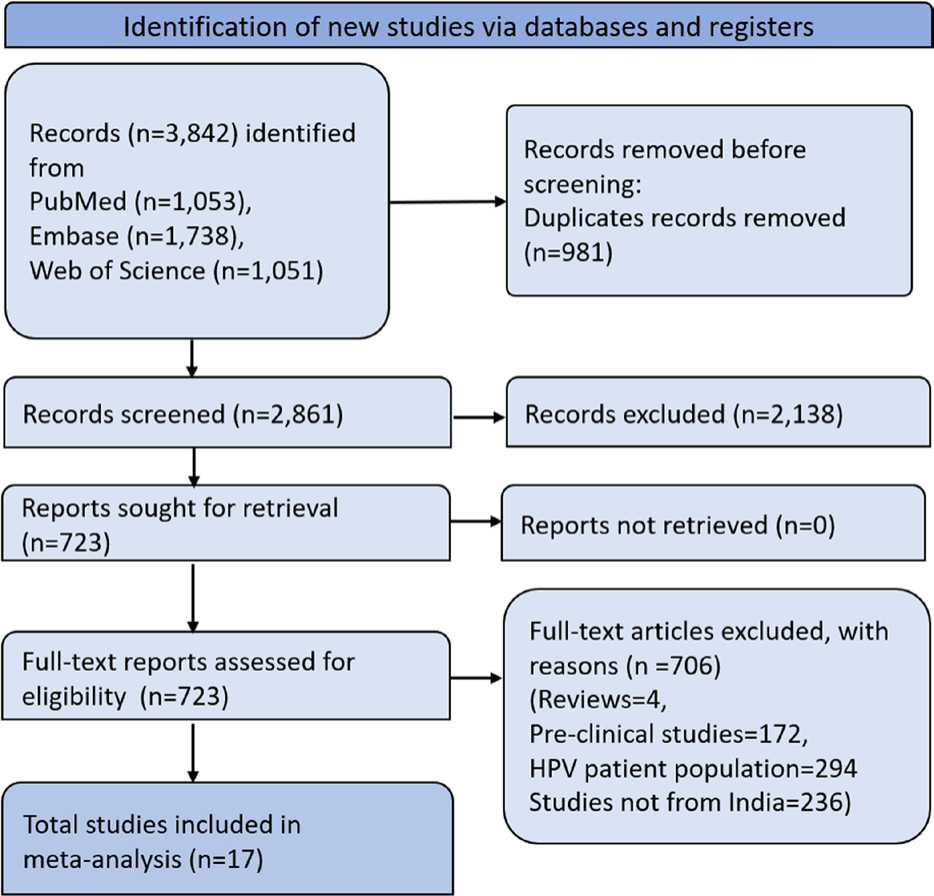
PRISMA flow chart representing the screening and selection of studies. PRISMA = Preferred Reporting Items for Systematic Reviews and Meta-Analyses.

The Preferred Reporting Items for Systematic Reviews and Meta-Analyses flow diagram presented in Figure 1 outlines the stages of the screening process and study selection for the systematic literature review. Initially, 3842 articles were identified. Duplicates amounting to 981 were subsequently removed, leaving 2861 records for screening. Of these, 2138 records were excluded during the screening phase, which led to 723 reports being retrieved for full-text assessment. All retrieved reports were evaluated for eligibility, and this evaluation resulted in the exclusion of 706 full-text articles for various reasons, such as being reviews, preclinical studies, involving HPV patient population, or relating to regions outside India. Ultimately, 17 studies met the eligibility criteria and were included in the meta-analysis.

### 3.2. Characteristics of included studies

Table 1 summarizes the characteristics of the included studies. These studies vary in design, including prospective observational,^[22,23]^ cohort,^[24]^ and cross-sectional studies.^[25–38]^ These studies encompass a wide range of Indian states and zones, from the southern regions of Tamil Nadu, Hyderabad, and Kerala^[23,31,34]^ to the northern states like Uttar Pradesh,^[37]^ and from the eastern state of Assam^[27]^ to the central and western states such as Maharashtra, Bhopal, and Gujarat.^[33,35,38]^ The participant age groups vary, with some studies specifying a mean age, such as 51.4 years^[25]^ and 70 years,^[38]^ while others do not. The diagnostic techniques used are primarily polymerase chain reaction (PCR)-based, including standard PCR and Nested PCR,^[27,32]^ with some studies also utilizing non-multiplex PCR.^[32]^ The sample sizes vary significantly, potentially impacting the robustness and generalizability of the results. Variability in HPV positivity rates is evident, reflecting the heterogeneity in cervical cancer cases across different Indian locales. The studies span an extensive period, allowing for an evaluation of changes in HPV prevalence over time. New Delhi is a significant contributor to the body of research,^[26,28]^ while Uttar Pradesh,^[37]^ Maharashtra,^[29,35]^ South India,^[31,34]^ Haryana, and Tamil Nadu^[34]^ also add valuable data from their respective regions. Studies from Andhra Pradesh,^[32]^ Kerala,^[31]^ Kolkata, Mumbai, Karnataka, Chandigarh, and Assam each contribute unique perspectives to the review. Moreover, a multi-regional study incorporates data from various states, providing a broader view of HPV prevalence.^[25]^ The quality of the studies, assessed by the modified Newcastle-Ottawa Scale, is rated as moderate to high, supporting the reliability of the findings in this review (Table S4, Supplemental Digital Content, http://links.lww.com/MD/N144).

**Table 1:** Main characteristics of the studies.

Study	Study design	State or regions	Zone	Age	Number of cervical cancer patients	Number of HPV-positive samples	Method of testing HPV
Baskaran 2015^[1]^	Prospective observational study	Tamil Nadu	South	30–65 years	67	63	PCR
Basu 2009^[2]^	Cross-sectional study	West Bengal, Karnataka, New Delhi, Maharashtra	Multiple	51.4 (mean)	278	232	PCR
Bhatla 2006^[3]^	Cross-sectional study	New-Delhi	North	NA	106	104	PCR
Das 2013^[4]^	Cross-sectional study	Assam	East	NA	107	105	Nested PCR
Gautam 2023^[5]^	Cross-sectional study	New-Delhi	North	NA	108	78	PCR
Gheit 2009^[6]^	Cross-sectional study	Maharashtra	Central	NA	168	113	PCR
Kumar 2021^[7]^	Cross-sectional study	Bihar	East	NA	96	96	PCR
Kuriakose 2020^[8]^	Cross-sectional study	Kerala	South	56 (mean)	114	90	PCR
Nagaraja 2023^[9]^	Cross-sectional study	Andhra Pradesh	South	NA	204	163	NM-PCR
Patel 2014^[10]^	Cross-sectional study	Gujarat	West	51.3 (mean)	52	31	PCR
Peedicayil 2006^[11]^	Cross-sectional study	Tamil Nadu	South	NA	119	113	PCR
Saranath 2002^[12]^	Cross-sectional study	Maharashtra	Central	NA	337	258	PCR
Sontakke 2019^[13]^	Cross-sectional study	Maharashtra	Central	NA	144	82	PCR
Srivastava 2021^[14]^	Cross-sectional study	Uttar Pradesh	North	21 (mean)	130	17	PCR
Thobias 2021^[15]^	Cohort study	Ahmedabad	West	NA	400	348	PCR
Sowjanya 2005^[16]^	Prospective observational study	Hyderabad	South	55 (median)	41	36	PCR-based line blot assay
Gupta 2022^[17]^	Cross-sectional study	Bhopal	South	31–70 years	58	48	Multiplex RT-PCR

NM-PCR = non-multiplex polymerase chain reaction, PCR = polymerase chain reaction.

### 3.3. Prevalence of HPV among cervical cancer patients

**Figure 2.**
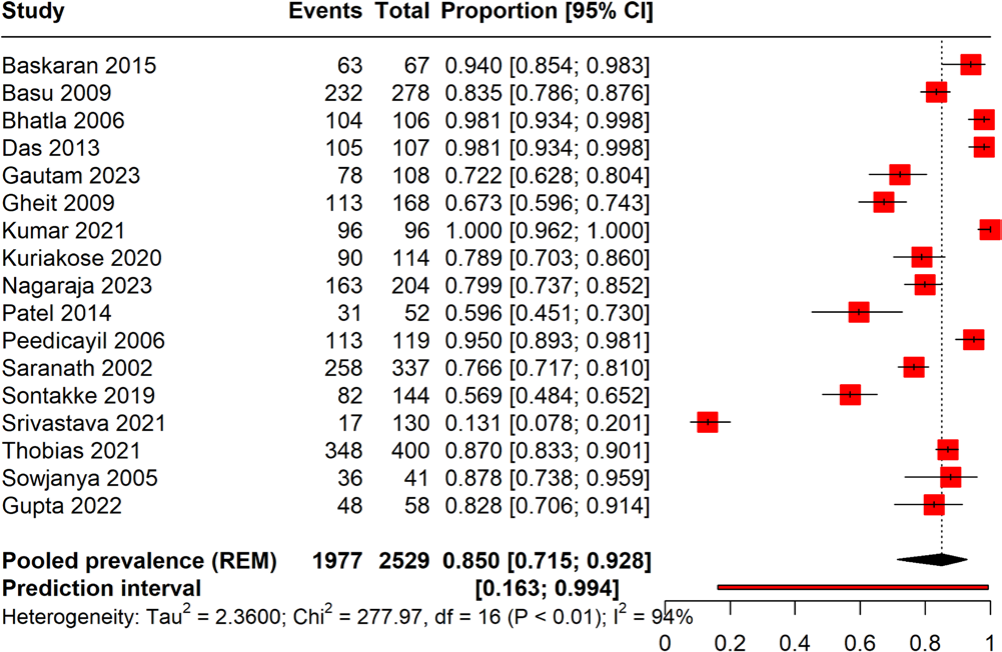
Forest plot illustrating the pooled prevalence of HPV positive in patients with cervical cancer.

The forest plot given in Figure 2 illustrates the prevalence of HPV among cervical cancer patients, derived from a meta-analysis of various studies ranging from the year 2005 to 2023. The total number of cervical cancer cases included in the analysis is 2529, with 1977 cases testing positive for HPV. This variation may be attributable to differences in study design, population, and diagnostic criteria for HPV, reflecting considerable heterogeneity as indicated by a high I² value of 95%. The pooled prevalence is estimated at 82% (95% CI: 71% to 92%), signifying a strong association between HPV and cervical cancer across studies. Notably, the plot reveals substantial precision disparities among studies, as evidenced by varying confidence interval widths. The prediction interval ranges widely from 16% to 99.4%, suggesting that future studies could report a broad spectrum of HPV prevalence among cervical cancer patients due to diverse factors such as geographic, population characteristics, and methodological approaches. The high I² (94%) value suggests that there is substantial variability in the study outcomes that may not be due to chance alone. The Chi² value and its associated *P*-value (<.01) further confirm significant heterogeneity across studies.

### 3.4. Subgroup analysis

**Figure 3.**
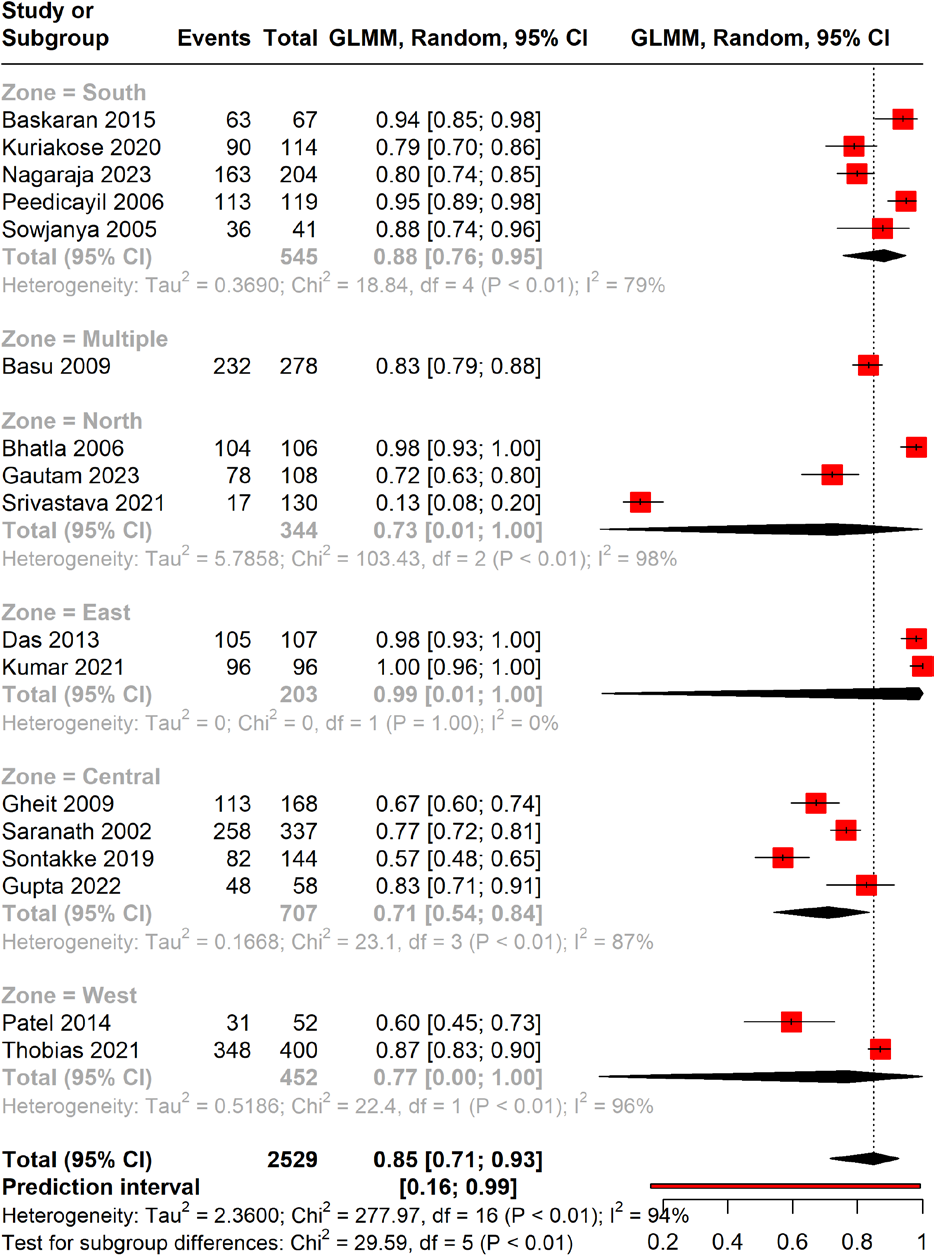
Forest plot representing the zone-wise subgroup analysis of prevalence of HPV in cervical cancer patients.

The forest plot illustrates the results of a subgroup analysis by geographical zone, assessing the prevalence of HPV among cervical cancer patients (Fig. 3). The analysis categorized studies into 5 distinct zones: South, Multiple, North, East, Central, and West. The pooled prevalence of HPV varied across zones, ranging from 71% in the Central zone to 99% in the East zone, with the overall pooled prevalence being 82% (95% CI: 62–93%). Notably, the heterogeneity within subgroups remained high, with I² statistics indicating substantial variability: 88% in the South, 73% in the North, 99% in the East, 71% in the Central, and 77% in the West. Moreover, the test for subgroup differences yielded a Chi² of 29.59 (df = 5, *P* < .01), which suggests significant differences in HPV prevalence across the geographical zones.

### 3.5. Sensitivity analysis

**Figure 4.**
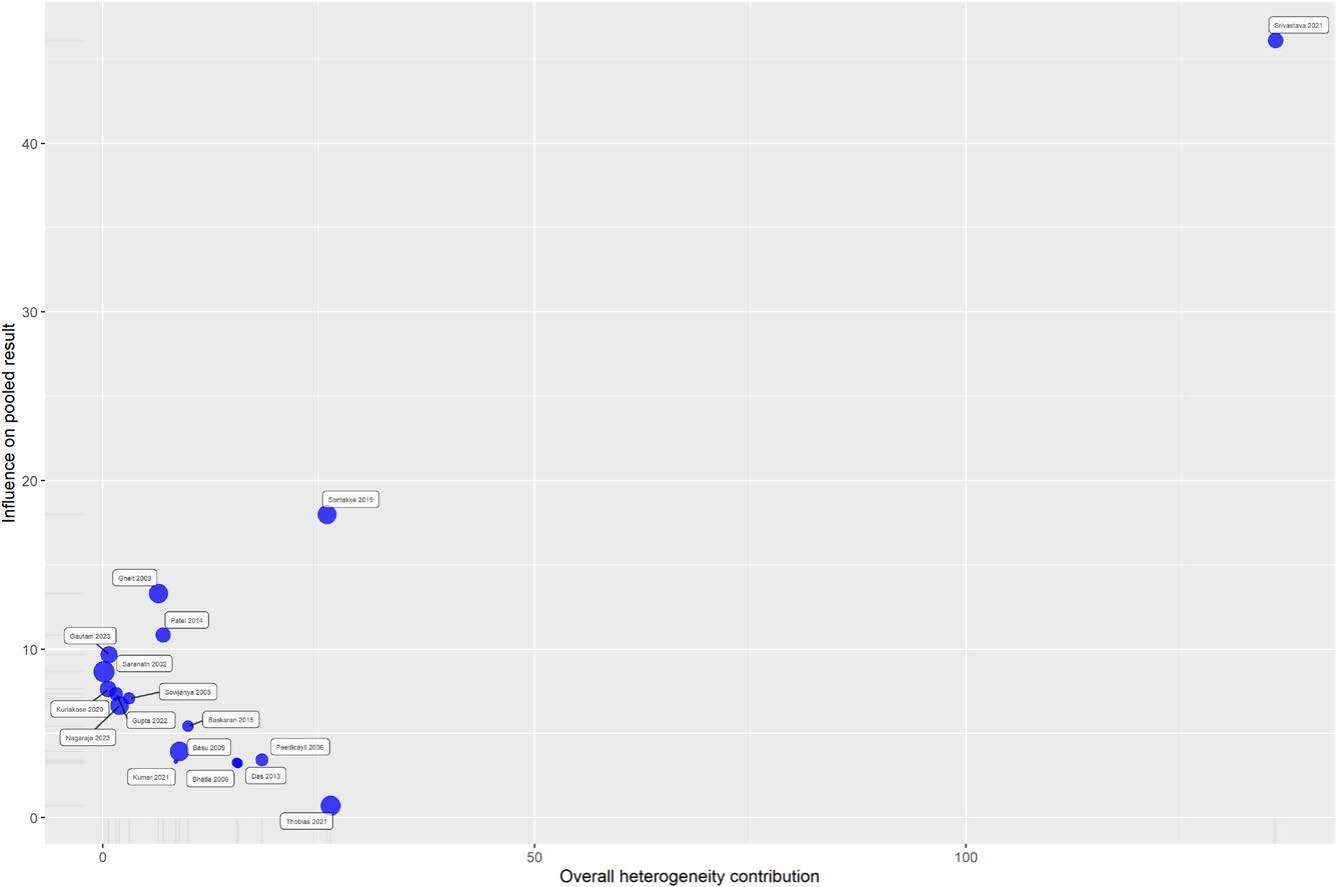
Baujat plot indicating studies affecting the result.

We performed several sensitivity analyses to evaluate the effect of individual studies on the overall result. We used a Baujat plot to assess the individual study contributions to the overall heterogeneity and to investigate potential sources of bias (Fig. 4). The horizontal axis of the plot quantifies the contribution of each study to the heterogeneity, and the vertical axis measures the influence of each study on the meta-analytic summary effect. Upon analysis, the Baujat plot revealed that the study by Srivastava et al^[37]^ displayed a substantial contribution to the overall heterogeneity as well as a pronounced influence on the pooled effect size, as indicated by its position in the upper right quadrant. Similarly, the study by Srivastava et al was also a notable contributor to heterogeneity, albeit with a lesser influence on the overall result. These findings suggest that the results reported by Srivastava et al^[37]^ may be outliers or possess unique characteristics that affect their weight in the meta-analysis. Factors such as larger sample sizes, methodological variances, or distinct population attributes may account for this observation. Influence diagnostics were utilized to assess the robustness of our meta-analysis. These diagnostics encompassed standardized residuals, DFFITS, Cook distance, covariance ratios, hat values, and study weights (Fig. S1, Supplemental Digital Content, http://links.lww.com/MD/N139). The analysis indicated variability in individual study influence, with some studies displaying large residuals and others showing substantial impact on the regression coefficients and fitted values. Deviations in covariance ratios and elevated hat values further highlighted studies with notable influence.

In order to assess the stability of our findings, we conducted a leave-one-out sensitivity analysis, where the pooled prevalence of HPV among cervical cancer patients was recalculated while sequentially omitting each study (Fig. S2, Supplemental Digital Content, http://links.lww.com/MD/N140). The analysis demonstrates that the omission of any single study from the meta-analysis does not result in substantial variations in the pooled prevalence, this consistency across sensitivity tests indicates a robust pooled estimate, suggesting that the meta-analytic conclusion is not unduly influenced by any individual study.

### 3.6. Reanalysis

**Figure 5.**
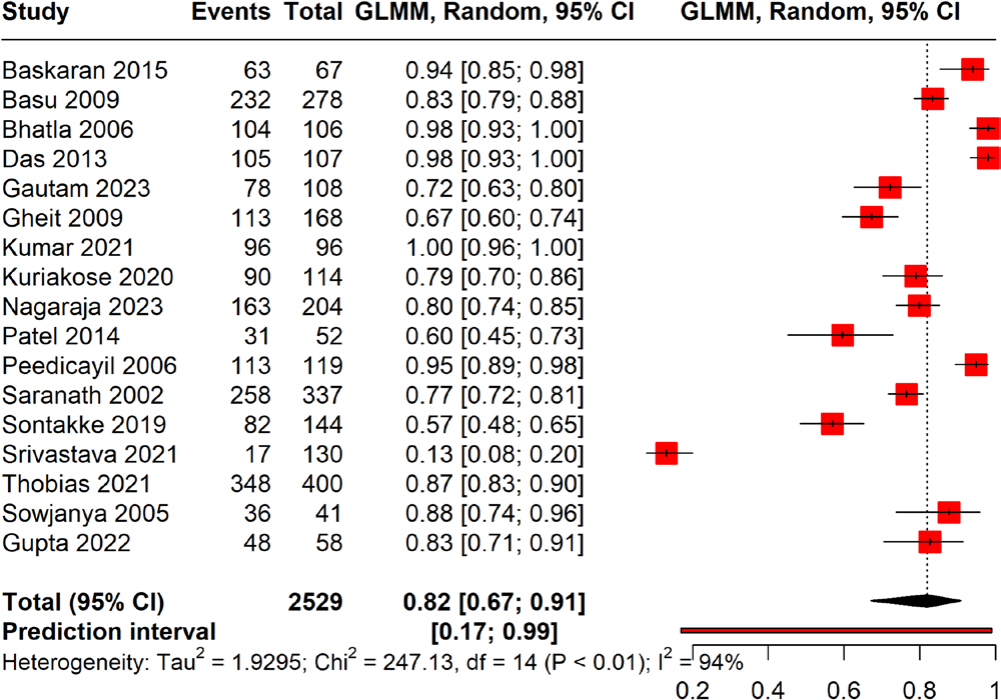
Forest plot showing the pooled prevalence of HPV in cervical cancer patients by reanalysis after removing the outliers.

Following the identification of 2 influential studies via a Baujat plot analysis, we conducted a reanalysis of our meta-analytic data on HPV prevalence among cervical cancer patients (Fig. 5). These studies were excluded to assess their impact on our initial findings. The recalculated pooled prevalence of HPV is now estimated at 82% (95% CI: 67% to 91%). Despite the exclusion of the influential studies, the prediction interval remains wide at 27% to 99%, reflecting the anticipated variability in future studies’ findings. The high level of heterogeneity, with an I² of 94%, persists, suggesting that factors beyond those captured by the removed studies contribute to the observed variance. These results reinforce the high prevalence of HPV among cervical cancer patients while highlighting the complexity and multifactorial nature of the underlying study heterogeneity.

### 3.7. Publication bias

**Figure 6.**
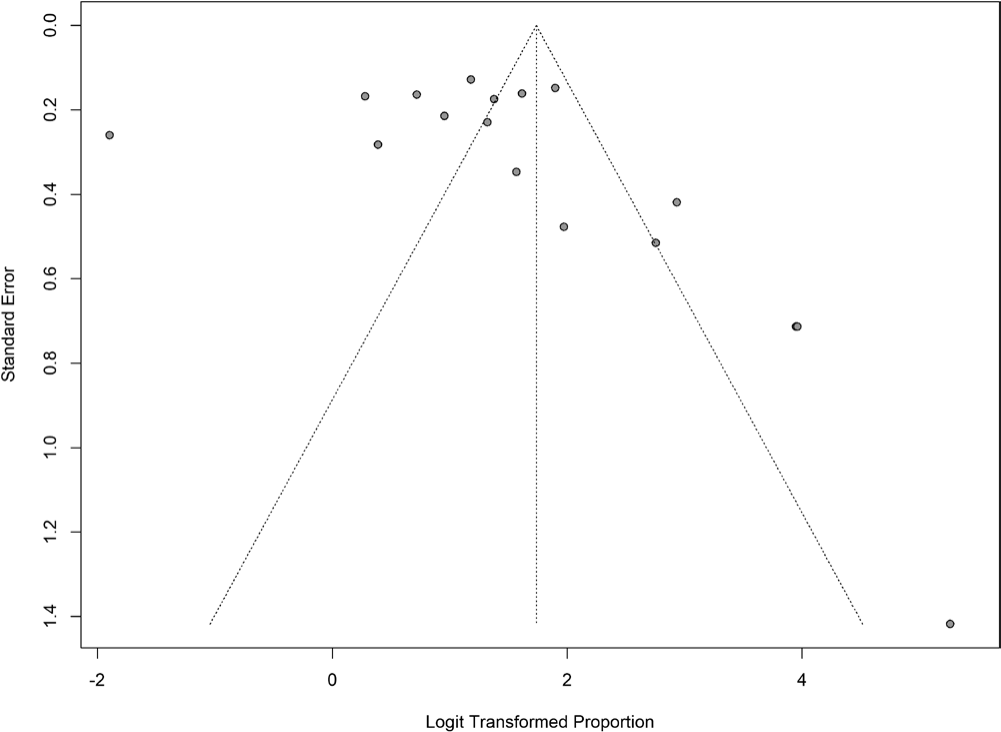
Funnel plot illustrating the presence of publication bias.

A funnel plot was constructed as a visual tool to explore the presence of publication bias (Despite the plot’s asymmetry hinting at potential bias, the Egger test yielded a *P*-value of .5994, suggesting no significant statistical evidence of publication bias (Fig. 6)). This discrepancy underscores the importance of cautiously interpreting asymmetry in funnel plots, as it may not necessarily denote bias but could arise from other sources of heterogeneity. Therefore, while the funnel plot’s shape indicates the possibility of missing studies or small-study effects, the Egger test provides reassurance that the meta-analysis findings are unlikely to be influenced by publication bias.

## 4. Discussion

To the best of our knowledge, this is the first systematic review and meta-analysis to evaluate the prevalence of HPV among cervical cancer patients. Our findings, based on 17 studies, indicate a high pooled HPV prevalence of 85% among cervical cancer patients, showing the significant role of HPV in the etiology of cervical cancer in this region. This significant finding not only highlights the predominant role of HPV in the etiology of cervical cancer in the region but also underscores the urgent need for focused public health interventions. The pronounced heterogeneity observed in our analysis, as indicated by an I² value of 95%, suggests a complex interplay of factors influencing HPV prevalence. These factors could range from regional variations in HPV type distribution to the diversity in diagnostic methodologies, study designs, and the demographic characteristics of the populations studied. Our subgroup analysis further illuminates this heterogeneity, revealing substantial differences in HPV prevalence across various Indian geographical zones. This variance in prevalence shows the importance of tailoring public health strategies to specific regional contexts to effectively combat HPV and its associated health burdens.

Our findings reveal significant regional variations in the prevalence of HPV among cervical cancer patients in India, underscoring the influence of diverse epidemiological factors. Factors such as differences in healthcare accessibility, public health policies, socio-economic status, and cultural practices across regions play a crucial role in shaping these disparities. For instance, regions with enhanced access to healthcare and higher socio-economic status often report higher rates of HPV screening and vaccination, which correlate with lower HPV prevalence rates. Conversely, areas with limited healthcare resources struggle with both higher HPV prevalence and cervical cancer rates. This correlation underscores the necessity of understanding and addressing the specific epidemiological factors that contribute to HPV prevalence to effectively tailor public health interventions. The findings highlight the importance of focusing on these regional epidemiological determinants rather than a generalized approach to vaccination and screening programs.^[39]^ Such targeted interventions are essential for the effective reduction of HPV prevalence and, by extension, cervical cancer incidence and mortality in India.

The previous meta-analysis explored HPV prevalence across various stages of cervical precancer and cancer, examining 5990 cases.^[40]^ It revealed that HPV prevalence was 85.4% in invasive cervical cancer (ICC) cases, 71.3% in CIN II–III or HSIL cases, 59.2% in CIN I or LSIL, and 34.8% in ASCUS cases. The most common genotype in ICC was HPV 16, found in 58% of cases, followed by HPV 18 at 16.5%. The study concluded that the integration of current HPV vaccines into national immunization programs and the establishment of comprehensive cervical screening strategies in Eastern Mediterranean countries could potentially prevent about 74.5% of cervical neoplasia cases. A meta-analysis reported that the overall HPV prevalence in cervical cancer cases in the Middle East and North Africa (MENA) region was 81% (95% CI, 70–90%).^[41]^ The highest rates were observed in the Maghreb at 88% (95% CI, 78–96%) and the lowest in Iran at 73% (95% CI, 62–83%). For individuals with abnormal cervical cytology in MENA, the prevalence was 54% (95% CI, 41–67%), peaking in Northeast Africa at 94% (95% CI, 91–96%) and dropping to 31% in the Levant (95% CI, 16–49%). Among the general population, HPV prevalence was 16% (95% CI, 14–17%), with the highest rates in Northeast Africa at 21% (95% CI, 7–40%) and the lowest in the Levant at 7% (95% CI, 2–14%). In Eastern Asia, the contribution of HPV52 and HPV58 to cervical intraepithelial neoplasia and invasive cancer was respectively 2.5 to 2.8 times and 3.7 to 4.9 times higher than in other regions, reported by another analysis.^[42]^ Our analysis contributes additional evidence from India, which is comparable to these results.

The public health implications of our findings are extensive and multi-faceted. First and foremost, they reinforce the crucial role of HPV vaccination programs, which need to be promoted more aggressively, especially in regions characterized by lower screening rates and higher incidences of cervical cancer. There is a clear and pressing need to increase awareness about HPV vaccination, particularly targeting its accessibility among underserved and high-risk populations. Our study also highlights the necessity for the establishment of standardized, high-quality cervical cancer screening programs. These programs should ideally be integrated with HPV vaccination initiatives and tailored to address the unique needs of different regions, ensuring that both rural and urban populations have equitable access to these services. Innovative approaches, such as the deployment of mobile health units, the involvement of community health workers, and the implementation of local educational campaigns, could be particularly effective strategies in increasing the coverage of both screening and vaccination.

Looking forward, future research should place a strong emphasis on longitudinal studies to evaluate the long-term impact of HPV vaccination on the incidence and mortality rates of cervical cancer. Such research is vital in understanding the broader implications of vaccination over time. Additionally, there is an urgent need to explore the various barriers that impede vaccination and screening, particularly in rural and socioeconomically disadvantaged communities. Understanding these barriers is crucial for developing targeted interventions that can effectively address these challenges. Future studies should also delve into the dynamics of vaccine acceptance, paying particular attention to the role of sociocultural factors in shaping health-seeking behaviors among different populations. As the landscape of HPV vaccines evolves with the introduction of vaccines covering additional strains, it is imperative to assess their efficacy specifically within the Indian demographic context. Moreover, the potential of emerging technologies and screening methods, such as self-sampling for HPV testing, should be explored. These technologies could play a significant role in overcoming existing barriers to screening, thereby broadening its reach and impact. The development and implementation of more sensitive and cost-effective diagnostic tools are also essential for facilitating earlier detection and treatment of cervical cancer.

Our study has some limitations that should be noted. The source of the high degree of heterogeneity observed in our meta-analysis (I² = 95%) was not fully elucidated. Despite conducting thorough subgroup and sensitivity analyses to explore potential sources of this variation, the underlying reasons for the heterogeneity remain partially unexplained. This suggests that there could be other unmeasured or unreported factors contributing to the variability in HPV prevalence among cervical cancer patients in India. The geographic coverage of the studies included in our analysis was not uniform. Some regions of India were not represented in the available literature, which could potentially skew the overall prevalence estimates and limit the generalizability of our findings. The lack of data from certain areas prevents a truly comprehensive understanding of the national landscape of HPV prevalence in cervical cancer patients. We were unable to perform subgroup analysis based on different HPV genotypes because several studies did not report this information. Additionally, there was potential overlap of genotypes within the same sample, and the reporting formats varied significantly across studies. This variability in data presentation and the lack of specific genotype information precluded a detailed subgroup analysis. Additionally, our study was limited to articles published in scientific databases, which may have excluded relevant studies not indexed in these databases. Such studies could hold valuable insights, especially from regions or subpopulations that are underrepresented in mainstream scientific literature. Given these limitations, there is a clear need for future studies in this area.

Research expanding the geographic scope within India to include underrepresented regions is essential to obtain a more accurate depiction of HPV prevalence among cervical cancer patients. Including clinical symptoms with individual variations in future research could offer more valuable insights for prospective researchers in the field of HPV and cervical cancer. Understanding how symptoms manifest differently among individuals can help identify high-risk populations and inform personalized screening and intervention strategies. Additionally, exploring the relationship between HPV genotype, clinical presentation, and disease progression may lead to more accurate risk stratification and improved patient outcomes. Overall, incorporating clinical symptomatology into research efforts can enhance our understanding of HPV-related cervical cancer pathogenesis and guide the development of more targeted and effective prevention and treatment approaches.

## 5. Conclusion

We found a high prevalence of HPV among cervical cancer patients in India, highlighting the critical need for enhanced HPV vaccination and cervical cancer screening programs. These findings underscore the importance of tailored public health strategies to address regional disparities in HPV prevalence and screening rates. Future research should focus on expanding geographic representation and exploring the factors contributing to the observed heterogeneity in HPV prevalence among cervical cancer patients.

## Acknowledgments

The authors acknowledge the Nested Knowledge team for providing the software access.

## Author contributions

**Conceptualization:** Prakasini Satapathy, Mahalaqua Nazli Khatib, Ahmad Neyazi, Laila Qanawezi, Shilpa Gaidhane, Quazi Syed Zahiruddin, Sarvesh Rustagi, Marwan Al-Hajeili, Altaf A. Abdulkhaliq, Ahmed Alsayyah, Hayam A. Alrasheed, Maha F. Al-Subaie, Nawal A. Al Kaabi, Ali A. Rabaan.

**Data curation:** Prakasini Satapathy, Laila Qanawezi, Sarvesh Rustagi.

**Formal analysis:** Prakasini Satapathy.

**Investigation:** Laila Qanawezi.

**Methodology:** Mahalaqua Nazli Khatib.

**Resources:** Saida Said.

**Software:** Saida Said.

**Writing – original draft:** Prakasini Satapathy, Mahalaqua Nazli Khatib, Ahmad Neyazi, Saida Said, Shilpa Gaidhane, Quazi Syed Zahiruddin, Sarvesh Rustagi, Marwan Al-Hajeili, Altaf A. Abdulkhaliq, Ahmed Alsayyah, Hayam A. Alrasheed, Maha F. Al-Subaie, Nawal A. Al Kaabi, Ali A. Rabaan.

**Writing – review & editing:** Prakasini Satapathy, Mahalaqua Nazli Khatib, Ahmad Neyazi, Shilpa Gaidhane, Quazi Syed Zahiruddin, Sarvesh Rustagi, Marwan Al-Hajeili, Altaf A. Abdulkhaliq, Ahmed Alsayyah, Hayam A. Alrasheed, Maha F. Al-Subaie, Nawal A. Al Kaabi, Ali A. Rabaan.

## Supplementary Material









**Figure:**
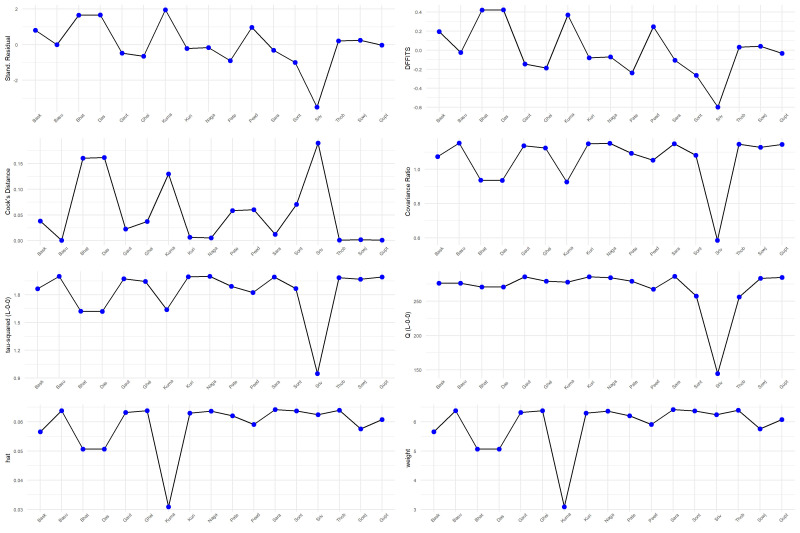


**Figure:**