# Supplementary material for: Prevalence of human papilloma virus among cervical cancer patients in India: A systematic review and meta-analysis
Source: Medicine (Baltimore). 2024 Aug 2;103(31):e38827. doi: 10.1097/MD.0000000000038827 (PMC11296450; doi:10.1097/MD.0000000000038827)
Supplement: Supplementary file 2 [file medi-103-e38827-s002.docx]

**Table S2.** Inclusion and Exclusion criteria

**Research Question: “What is the prevalence of HPV among patients with cervical cancer in India?”**

| **Inclusion** | | **Exclusion** |
| --- | --- | --- |
| **Participants** | - Patients with cervical cancer | HPV patient population |
| **Intervention/Exposure** | - Cervical cancer of any stage | Other type of cancers |
| **Outcome** | - No. of patients diagnosed with HPV among total cervical cancer patients (prevalence) |  |
| **Study Designs** | Cross-sectional, and cohort studies | Letter to editor,  Commentaries,  Qualitative studies, Abstract only, Case series, case reports, reviews, Discussion papers |
|  | Only published articles from India in English language restriction till 07^th^ of December 2023 | Unavailable full-text articles |
