# Supplementary material for: Prevalence of human papilloma virus among cervical cancer patients in India: A systematic review and meta-analysis
Source: Medicine (Baltimore). 2024 Aug 2;103(31):e38827. doi: 10.1097/MD.0000000000038827 (PMC11296450; doi:10.1097/MD.0000000000038827)
Supplement: Supplementary file 3 [file medi-103-e38827-s003.docx]

**Table S3.** The adjusted search terms as per searched electronic databases [as of 07.12. 2023]

| Database | Search Query | Results |
| --- | --- | --- |
|  | | |
| PubMed | ("Human Papillomavirus"[All Fields] OR "HPV"[All Fields]) AND ("Cervical Cancer"[All Fields] OR "Cervical Neoplasms"[All Fields] OR "Cervix Cancer"[All Fields] OR "Cervix Neoplasms"[All Fields]) AND (India) | **1,053** |
|  | | |
| Embase | ('human papillomavirus' OR 'HPV') AND ("Cervical Cancer" OR "Cervical Neoplasms" OR "Cervix Cancer" OR "Cervix Neoplasms") AND India | 1,738 |
|  | | |
| Web of Science | ('human papillomavirus' OR 'hpv') AND ("Cervical Cancer" OR "Cervical Neoplasms" OR "Cervix Cancer" OR "Cervix Neoplasms") AND India (All fields) | 1,051 |
