# Supplementary material for: Prevalence of human papilloma virus among cervical cancer patients in India: A systematic review and meta-analysis
Source: Medicine (Baltimore). 2024 Aug 2;103(31):e38827. doi: 10.1097/MD.0000000000038827 (PMC11296450; doi:10.1097/MD.0000000000038827)
Supplement: Supplementary file 4 [file medi-103-e38827-s004.docx]

**Table S4.** Quality assessment using modified Newcastle-Ottawa Scale

| **Sl.no** | **Study** | **Representativeness** | **Sample size 200** | **Cervical definition** | **Ascertainment of HPV diagnosis/detection** | **Total** |
| --- | --- | --- | --- | --- | --- | --- |
|  | Baskaran 2015 | 2 | 0 | 1 | 1 | 4 |
|  | Basu 2009 | 2 | 1 | 1 | 1 | 5 |
|  | Bhatla 2006 | 2 | 0 | 2 | 1 | 5 |
|  | Das 2013 | 2 | 0 | 1 | 1 | 4 |
|  | Gautam 2023 | 2 | 0 | 2 | 1 | 5 |
|  | Gheit 2009 | 2 | 0 | 2 | 1 | 5 |
|  | Kumar 2021 | 2 | 0 | 2 | 1 | 5 |
|  | Kuriakose 2020 | 2 | 0 | 2 | 1 | 5 |
|  | Nagaraja 2023 | 2 | 1 | 1 | 1 | 5 |
|  | Patel 2014 | 2 | 0 | 1 | 1 | 4 |
|  | Peedicayil 2006 | 2 | 0 | 2 | 1 | 5 |
|  | Saranath 2002 | 2 | 0 | 2 | 1 | 5 |
|  | Sontakke 2019 | 2 | 0 | 1 | 1 | 4 |
|  | Srivastava 2021 | 2 | 0 | 1 | 1 | 4 |
|  | Thobias 2021 | 2 | 1 | 2 | 1 | 6 |
|  | Sowjanya 2005 | 2 | 0 | 1 | 1 | 4 |
|  | Gupta 2022 | 2 | 0 | 2 | 1 | 5 |
